# Implementation of a coach-supported mHealth intervention for dementia prevention in China: a qualitative study among Chinese participants and coaches in the PRODEMOS trial

**DOI:** 10.7189/jogh.15.04036

**Published:** 2025-03-28

**Authors:** Jinxia Zhang, Marieke P Hoevenaar-Blom, Xuening Jian, Haifeng Hou, Siqi Ge, Carol Brayne, Esmé Eggink, Melanie Hafdi, Mingyue He, Guohua Wang, Wenzhi Wang, Wei Zhang, Yueyi Yu, Yixuan Niu, Jihui Lyu, Libin Song, Wei Wang, Youxin Wang, Eric P Moll van Charante, Manshu Song, PRODEMOS study group, Hongmei Liu, Bin Jiang, Hong Wang, Haixin Sun, Xiaojuan Ru, Dongling Sun, Huimin Lu, Cancan Li, Xiaoyu Zhang, Hao Wang, Tenghong Lian, Weijiao Zhang, Wenjing Zhang, Jing Qi, Jinghui Li, Huiying Guan, Dongmei Luo, Weijia Zhang, Hao Yue, Zijing Zheng, Yan Song, Xiaosheng Meng, Sirui Zhu, Qiang Zeng, Huangdai Yang, Yanyan Tang, Tianqi Tao, Dongmei Jia, Mo Li, Wenjie Li, Haiyan Mu, Wenjing Jiang, Wenchao Gao, Yueqing Hu, Xizhu Xu, Yichun Zhang, Dong Li, Hui Yuan, Xiaojing Yin, Liang Dong, Xiaoyan Ye, Xi Wei

**Affiliations:** 1 Beijing Key Laboratory of Clinical Epidemiology, School of Public Health, Capital Medical University, Beijing, China; 2 School of Medical and Health Sciences, Edith Cowan University, Perth, Australia; 3 Department of General Practice, Amsterdam UMC, University of Amsterdam, Amsterdam, the Netherlands; 4 Department of Public and Occupational Health, Amsterdam UMC, University of Amsterdam, Amsterdam, the Netherlands; 5 School of Public Health, Shandong First Medical University & Shandong Academy of Medical Sciences, Jinan, Shandong, China; 6 Department of Neuroepidemiology, Beijing Neurosurgical Institute, Beijing, China; 7 Cambridge Public Health, University of Cambridge, Cambridge, UK; 8 Department of Neurology, Amsterdam UMC, University of Amsterdam, Amsterdam, the Netherlands; 9 Centre for Cognitive Neurology, Department of Neurology, Beijing Tiantan Hospital, Capital Medical University, Beijing, China; 10 The Second Affiliated Hospital of Shandong First Medical University, Tai’an, Shandong, China; 11 Innovation Centre for Neurological Disorders, Department of Neurology, Xuanwu Hospital, Capital Medical University, Beijing, China; 12 Department of Geriatrics, The Second Medical Centre & National Clinical Research Centre for Geriatric Diseases, Chinese PLA General Hospital, Beijing, China; 13 Centre for Cognitive Disorders, Beijing Geriatric Hospital, Beijing, China; 14 Comvee Research Institute, Fuzhou Comvee Network & Technology, Fuzhou, China; 15 Chemistry and Chemical Engineering Guangdong Laboratory, Shantou, Guangdong, China; 16 Institute for Glycome Study and The First Affiliated Hospital, Shantou University Medical College, Shantou, Guangdong, China

## Abstract

**Background:**

Modifiable risk factors have been linked to 45% of dementia cases. Mobile health (mHealth) interventions targeting lifestyle-related risk factors with remote coaching have the potential to reach underserved high-risk populations globally. To date, little is known about the implementation of such interventions in China.

**Methods:**

Fifty semi-structured interviews were conducted with 14 participants and 11 health coaches involved in the PRODEMOS trial. This trial investigated whether a coach-supported mHealth application intervention can reduce dementia risk in people aged 55–75 years with multiple risk factors. Interviews were conducted three months and 12–18 months into the intervention, focusing on implementation outcomes among Chinese participants using thematic analysis.

**Results:**

Participants found the PRODEMOS app easy to use and remote coaching convenient, although coach responses were sometimes perceived as slow due to not logging into the mHealth platform simultaneously, thus delaying text chat communication. The intervention's appropriateness was shaped by its effectiveness in enhancing health awareness and meeting participants’ needs. Feasibility depended on integration into daily routines, participant progress, partner support, coach attention, smartphone literacy, and time availability. Challenges for the coaches included remote motivational interviewing and sustained participant-coach engagement, influenced by participant-coach relationships, social environment, and the COVID-19 pandemic. Participants generally adhered to goals, but fidelity varied. Integration into primary care was endorsed.

**Conclusions:**

This first qualitative study of the Chinese arm of the PRODEMOS intervention demonstrates that it is an acceptable and implementable approach for promoting lifestyle changes in individuals at increased risk of dementia. While coaching is crucial for sustained engagement, it presents challenges when delivered remotely. Despite significant variability in participants' adherence, positive feedback underscores its potential for integration into primary care and large-scale implementation, provided issues with coaching and engagement are addressed. These findings offer valuable insights for practitioners and policymakers seeking to incorporate mHealth solutions into public health strategies for dementia prevention.

**Registration:**

PRODEMOS: ISRCTN15986016.

Over the coming decades, a steep rise in the number of people living with dementia in ageing societies, such as China, is expected [1]. Up to 45% of dementia has been estimated to be associated with 12 risk factors, including less education, smoking, physical inactivity, hypertension, obesity, diabetes, and excessive alcohol consumption [2]. However, randomised controlled trials (RCTs) addressing these risk factors have yielded inconsistent results [3]. It is essential to identify new strategies and factors that influence successful implementation of preventive interventions in populations that are underserved and at increased risk.

Mobile health (mHealth) refers to the use of mobile phones and wireless technology in health care, with much reported potential to deliver affordable and easily accessible health care support to diverse populations [4]. It has shown some promising effects in improving adherence to a healthy lifestyle [5]. However, research findings demonstrate heterogeneity in implementation [6], which is influenced by factors such as smartphone literacy, health expectations, and uptake in volunteer cohorts, which do not represent full populations. These factors potentially shape the acceptability of interventions [7,8]. The appropriateness of interventions is further affected by user-friendliness and customised design of the mobile app [9,10]. Moreover, systematic reviews on mHealth interventions for managing lifestyle-related risk factors for dementia and cardiovascular disease in adults suggest that a blended approach, combining a digital health platform with human support, improves motivation and foster habit formation, thereby promoting sustainability. However, while studies on the efficacy of mHealth interventions with remote coaching are relatively common in high-income countries (HICs), they remain scarce in low- and middle-income countries (LMICs) and among populations with low socioeconomic status (SES) within HICs [10,11]. This gap inspired the conception and conduct of the ‘PRevention Of DEmentia using MObile phone applicationS (PRODEMOS)’ trial (Trial registration number ISRCTN15986016) [12,13]. The PRODEMOS study is a multinational, prospective, randomised, open-label blinded endpoint (PROBE) clinical trial with a 12–18-month intervention and follow-up period. It was designed to investigate the effectiveness and implementation of a coach-supported mHealth intervention to optimise self-management of dementia risk factors in individuals aged 55–75 years at increased risk of dementia. It focused on low socioeconomic status (SES) in the UK and the general population in greater Beijing, with the aim to reduce overall dementia risk.

Few studies have been conducted within Chinese older adult populations [8], with one indicating a crucial role for self-efficacy in engaging participants in mHealth interventions for chronic non-communicable diseases. Additionally, questionnaire-based studies on Chinese populations have highlighted how the ability to use electronic devices and trust levels impact the feasibility of interventions [14]. Notably, existing research in Chinese populations prior to this study has mostly focused on managing specific diseases, such as coronary heart disease, obesity, diabetes mellitus, stroke [11], and chronic kidney disease [11], or individual risk factors (*e.g*. physical inactivity) [15]. This research gap, combined with the tendency of previous studies to focus primarily on participants while neglecting the roles of health care professionals and patient supporters, highlights a need for evidence on the implementation of coach-supported mHealth approaches for both cardiovascular disease and dementia prevention.

Therefore, the current study aims to explore the implementability of the PRODEMOS intervention – an interactive, health coach-supported mHealth intervention targeting dementia risk factors [12,13] – in a middle-income country context, with a focus on China. This evaluation focused on qualitative interviews with Chinese intervention participants and their coaches, assessing key indicators of successful implementation [16], including the appropriateness, acceptability, feasibility, sustainability, and fidelity of the blended mHealth intervention.

## METHODS

### Study design and setting

The PRODEMOS trial was previously described [12,13]. In short, individuals aged 55 to 75 years, of low SES in the UK and of any SES in China, with two or more dementia risk factors and owning a smartphone, were eligible for inclusion. Exclusion criteria included dementia, cognitive impairment, conditions impeding an 18-month follow-up, smartphone illiteracy, and severe visual impairment. Participants were randomised between the intervention (an interactive app to improve lifestyle behaviours with coach support) and control condition (sham app without coach support) to assess the effect on dementia risk [12,13].

The PRODEMOS app was designed to support participants with sufficient digital literacy, defined as the ability to send a message using a smartphone [17]. Participants in the intervention group received the interactive PRODEMOS app, which facilitated self-management of seven dementia risk factors (overweight, unhealthy diet, insufficient physical activity, smoking, hypertension, dyslipidaemias, and diabetes) with remote coach-support over a period of 12 to 18 months. The health coaches included in China were four nurses, a health management specialist, a PhD candidate, three master’s students, and two undergraduate students. All coaches underwent specialised training in motivational interviewing (MI) [18] techniques before participating in the PRODEMOS trial and adhered to a coaching protocol aligned with current risk factor management guidelines [13]. Through the interactive app, intervention participants could set personalised goals to improve their lifestyle, following the SMART (Specific, Measurable, Achievable, Realistic, and Time-bound) principle. They received automatic reminders to enter measurements related to their goals into the app (*e.g*. number of steps, blood pressure, adherence to medication), and could view trends over time, facilitating progress monitoring. The app also provided access to (tailored) educational materials on lifestyle-related dementia risk factors and allowed intervention participants to receive personalised feedback from their coaches through the computer-based coach portal of the mHealth platform, specifically designed for the PRODEMOS trial. Coaches initially guided intervention participants either in person or via video call during the baseline visit, with all subsequent communication (remote coaching) conducted through the app's messaging feature (text and voice chat functionality). An explanatory animation video, accessible through the app’s library, was built in the app to demonstrate the main functionalities of the app and help participants fully utilise all features of the app. Further details about the design, development, interface, and user experience of the PRODEMOS app's functionalities were described previously [12,13,17].

This qualitative study included two rounds of semi-structured interviews with 14 Chinese intervention participants and all 11 health coaches involved in the Chinese part of the multinational PRODEMOS trial, recruited from seven hospitals (trial centres) in Beijing and Tai’an, China. Participants were purposively sampled from the intervention group based on study centre, age, gender, educational background, history of CVD and/or diabetes, and number of dementia risk factors to obtain a diverse sample. Coaches from all trial centres in China, representing diverse ages, genders, educational backgrounds, and professions, were included in the study. The first round of interviews took place approximately three months into the intervention (denoted as M3), while the follow-up interviews (Mfinal) were conducted 12 to 18 months into the intervention (*i.e*. one week after their final assessments), involving all participants and coaches who had taken part in the initial round of interviews.

### Data collection

From October 2022 to May 2023, two rounds of semi-structured interviews were conducted either in-person or via video calls by a female researcher (JZ), who possessed three years of experience in conducting qualitative interviews and had no prior connection with the interviewees. The first round of interviews followed an interview guide, focusing on participants' initial motivation, as well as the appropriateness, acceptability, feasibility, sustainability, and fidelity of the intervention (Appendix S1 in the **Online Supplementary Document**). The second round of interviews followed a modified interview guide with more emphasis on the sustainability and fidelity of the intervention (Appendix S2 in the **Online Supplementary Document**). Interviewees were also queried about their perspectives on coach-supported self-management and potential future perspectives for use inside and outside the health care context. The interview guides were iteratively adapted throughout the data collection phase.

All interviews were recorded and transcribed verbatim. To guarantee the accuracy of English translation, the original transcripts in Chinese were first translated into English, and then back translated into Chinese for verification. Data analysis was conducted on the English transcripts. Interviews lasted between 40 to 70 minutes, with a mean length of 56 minutes.

Six researchers (JZ, XJ, HH, MS, MPH, and EPMvC) engaged in regular video meetings to discuss the coding and thematic analysis together. They are all members of the PRODEMOS study group and have qualitative research experience. We adhered to the consolidated criteria for reporting qualitative research (COREQ) guidelines [19] to enhance the clarity and reproducibility of our reported findings.

### Qualitative analysis

We conducted thematic analysis following the six phases as described by Braun and Clarke [20].

1. Familiarisation with the data. After validating the transcripts and their English translation, XJ and MS shared the interview transcripts with MPH and EPMvC. Following each set of interviews, the researchers thoroughly reviewed and discussed all transcripts to ensure a comprehensive understanding of their content.

2. Generating initial codes. JZ and HH independently conducted initial coding using MAXQDA software. After completing the coding for one set of interviews, they compared and discussed their results to reconcile disparities and develop a refined set of codes. They summarised, distilled, and classified each sentence under one of the implementation outcomes previously delineated by Peters et al. [16] to generate initial codes that captured the essence of the corresponding data segments. For example, if participants mentioned needing to look after their grandchildren or a hospitalised spouse, thus being unable to find time for physical exercise, we coded this as ‘Feasibility>Lifestyle change>Barriers>Care for child or hospitalised spouse’. If a sentence fitted multiple implementation outcomes, we categorised it under each relevant outcome.

3. Searching for themes. We organised the material as much as possible under the chosen implementation outcomes while also identifying additional themes when certain elements did not align well with these outcomes. In MAXQDA, we used mind-mapping to create a thematic tree that grouped similar codes into themes and visualised connections to explore both pre-specified outcomes and emerging themes.

4. Reviewing themes. JZ, XJ, HH, MS, and MPH reviewed all transcripts to ensure that the data appropriately aligned with the pre-specified and additional themes.

5. Defining and naming themes. We further refined each theme to capture its main idea, generated clear definitions and names, and ensured that each theme remained distinct while fitting within the study’s broader conceptual framework.

6. Producing the report. JZ proposed narratives for each theme, which MS, MPH, and EPMvC discussed through video meetings. JZ and MS identified illustrative examples. XJ translated them into English, and MS validated the translations. We incorporated the final themes into the Results section, supporting them with direct quotes from interviewees to substantiate the findings.

To enhance the reliability of the findings, we also translated the codes into English in MAXQDA. Multiple researchers (JZ, XJ, HH, MS, MPH) with expertise in qualitative research and involvement in the PRODEMOS trial validated the codes, identified themes, and interpreted the analysis. We cross-checked each step against the original transcripts, and we discussed and resolved any discrepancies through consensus.

### Definition of the implementation outcomes

Implementation outcomes were defined after Peters et al. [16].

‘Appropriateness’ refers to the perceived relevance or fit of the intervention for the target population, setting, or issue being addressed. In this study, it relates to how well the blended mHealth intervention (PRODEMOS app combined with remote health coaching) aligns with the participants' needs for managing lifestyle-related dementia risk factors and whether it is applicable to their lifestyle.

‘Acceptability’ refers to the degree to which the intervention is satisfactory or agreeable to participants and coaches. In this study, it relates to the participants' and coaches' satisfaction with the app, its functionalities, and the coaching process, including whether they found the intervention agreeable and engaging.

‘Feasibility’ refers to the practicality and ease of implementing the intervention within the given context. This includes assessing whether the app and coaching methods could be successfully delivered, considering factors like technological access, ease of use, and the ability of both participants and coaches to engage with the intervention.

‘Sustainability’ refers to the extent to which the intervention can be maintained and integrated into routine practice over time. This includes assessing whether the intervention continues to be used by participants until the study ends.

‘Fidelity’ refers to the degree to which the intervention was delivered as intended, according to the original protocol and design. In this study, it involves evaluating whether the health coaches adhered to the motivational interviewing (MI) techniques, followed the structured coaching protocol, and maintained the integrity of the intervention throughout its implementation, and whether participants adhered to the intervention by using the app, and working on their lifestyle.

## RESULTS

### Description of interviewees

Fourteen (70%) of 20 eligible participants and all coaches who were invited for the interviews responded. We conducted 50 interviews with 14 participants aged 56-71 years, with 43% (6/14) having an education level of junior high school and below, 57% (8/14) having four or more risk factors, and 11 coaches aged 22–58 years, with 27% (3/11) working full-time. They were recruited from each of the seven participating trial centres, covering various Chinese communities. The characteristics of the participants and coaches are shown in **Table 1**, with more details of the interviewees provided in Table S1–2 in the **Online Supplementary Document**. One of the participants and one of the coaches who had dropped out of the trial seven and eight months into the intervention respectively also attended the second-round interview. One couple was also included, with each individual interviewed separately. Fifty percent (7/14) of the participants and 91% (10/11) of the coaches were women.

**Table 1:** Characteristics of the intervention participants (n = 14) and health coaches (n = 11)

Intervention participants	n (%)
**1.Participant characteristics**	
Age in years, median (range)	63 (56–71)
Gender (female)	7 (50)
Retired (yes)	9 (64)
History of CVD (yes)	5 (36)
History of diabetes (yes)	2 (14)
Education level	
*Junior high school and below*	6 (43)
*Senior high school and above*	8 (57)
Living situation	
*With spouse only*	9 (64)
*With spouse and other family*	5 (36)
No. of risk factors*	
*2*	1 (7)
*3*	5 (36)
*4 or more*	8 (57)
Region	
*Beijing (large city)*	11 (79)
*Tai’an*† *(small city)*	3 (21)
**2.Study characteristics**	
First FU duration in months, median (range)	2.9 (2.6–3.1)
Second FU duration in months, median (range)	12.9 (12.1–16.1)
Partner participating in the study (yes)	3 (21)
Login frequency per month, median (range)	19.9 (2.9–78.1)
**Health coaches**	
Age in years, median (range)	28 (22–58)
Gender (female)	10 (91)
Full-time (yes)	3 (27)
Occupation	
*Nurse*	4 (36)
*Health management specialist*	1 (9)
*PhD candidate*	1 (9)
*Master’s student*	3 (27)
*Undergraduate student*	2 (18)
Department or specialty	
*Neurology*	4 (36)
*Cognitive disorders*	4 (36)
*Internal medicine*	2 (18)
*Medical centre (health management)*	1 (9)
No. of participants coached (M3)‡, median (range)	10 (5–83)
No. of participants coached (Mfinal)§, median (range)	18 (7–89)

CVD – cardiovascular disease, FU – follow-up

*Risk factors include physical inactivity, active smoking, hypertension, cardiovascular disease, diabetes mellitus, dyslipidaemia, obesity, and depression.

†A prefectural city in Shandong province, China.

‡M3 – at approximately three months into the PRODEMOS intervention.

§Mfinal – at 12 to 18 mo into the PRODEMOS intervention.

### Motivation to participate in the PRODEMOS intervention

Most participants were driven by a concern for their physical well-being, specifically aiming to enhance their physical metrics and prevent health issues proactively. About half of the participants joined the current study due to concerns about dementia risk, particularly seeking information on prevention strategies.

*I take my health more seriously now. Last year, my brother died suddenly at the age of 66, which was a big blow for me. So, I thought that we should be kinder and more responsible to ourselves. –* Participant (P) 10, M3

*I'm trying to understand how to prevent dementia, which is something that scared me because I didn't know how to handle it. So, I'm eager to learn more about it. –* P5, M3

Coaches noticed that participants with a higher level of education and awareness demonstrated stronger motivation to engage in the intervention.

*I think participants with high education levels may have more medical knowledge and be more concerned about their health … They read the education materials more carefully because they know a lot and want to learn more. –* Coach (C) 10, M3

Some participants cited a desire to avoid burdening their children in case of illness as their motivation. Additionally, others were motivated by the offer of a complimentary physical examination, the opportunity to contribute to research, or an invitation from their doctor.

*My classmates suffer from Alzheimer disease, the pressure in the family, both financially and spiritually, is great. –* P2, M3

*At that time, I heard that the hospital was conducting a project and needed to recruit participants. I thought if I were one of the participants, I would also contribute to the research. –* P12, Mfinal

*My doctor told me that it would promote health and be good for my body. That's why I participated. –* P13, M3

### Implementation outcomes of the PRODEMOS intervention

The implementation outcomes of the PRODEMOS intervention illustrated by 60 quotes from both coaches’ and participants’ interviews are presented in **Table 2** and **Table 3**. We explored various aspects such as the use of the app, lifestyle change, health coaching (including MI), and remote coaching to evaluate all implementation outcomes separately.

**Table 2:** Appropriateness, acceptability, and feasibility of the PRODEMOS intervention

	Intervention participants		Coaches	
**Themes**	**Subthemes**	**Quotes**	**Subthemes**	**Quotes**
**Appropriateness**				
App/coach platform	Raise health awareness and promote lifestyle change	*Now that all kinds of knowledge are flying around on WeChat and it's hard to tell what's true and what's not, so having a channel to get scientific knowledge like this gives me peace of mind. (P5, Mfinal)*	Valuable guidance through educational resources	*The platform provides a lot of education materials for support of health management (of the participants). For me, sending education materials and reviewing/going over statistical graphs of trends in measurements (displayed in the platform), etc. helped me a lot in the participants’ health management. (C8, M3)*
		*Setting goals is a boost to improve exercise and diet … After setting a goal of increasing the number of steps, I want to walk 10 000 steps a day. (P7, M3)*		
Health coaching	Relevant for addressing needs	*Health coaching is very important to me. I was trying to lose weight at that time when I participated in the project. The coach advised me to eat less staple food and motivated me to do physical activity every day. Absolutely, it meets my needs. (P9, Mfinal)*	Motivational interviewing enhanced intrinsic motivation	*It's quite helpful to make participants aware of health-related issues and enhance their internal motivation to make healthier choices, and then I can help them, pay attention to them, and give them confidence in changing to a healthy lifestyle. (C7, Mfinal)*
		*My coach told me that when you do more exercise, don't engage in heavy exercise activities, but do what you can. I strongly agree with his advice. I have been exercising regularly throughout the year without engaging in high-intensity exercise, and it has made me feel relaxed. (P11, Mfinal)*		
	NA		Customised guidance, like a friend	*I feel that when communicating with participants, I should first ask them about their situation, not like a doctor treats patients, but like a friend… It felt that participants would be more willing to reply to or interact with me. (C10, Mfinal)*
	Preference for the combination of a coach and the app	*I believe a combination of both approaches is more effective. The coach can continuously demand and help me to set goals, and I must strive to attain them. This acts as a motivational push for me, making me feel more accomplished. The app can provide valuable knowledge and aid in achieving these goals. (P2, Mfinal)*	Combination of a coach and the app is more effective	*A participant always found it too difficult to quit smoking. Then I always encouraged him to start with a small goal. Then he became more convinced, especially after he checked education materials in the app about how smoking causes arteriosclerosis, coupled with the communication between us now and then. (C5, M3)*
Lifestyle change	Incorporation into daily routines	*It will be the same situation (maintaining a healthy lifestyle) not to say a year or so, probably ten years later because I have got into the habit. The (PRODEMOS) program is really quite convenient, and I'm a bit dependent on it now. (P5, Mfinal)*	Incorporation into daily routines	*A participant who needs to look after his wife with Parkinson at home all day. As such, I motivated him to go walking while taking his wife on days out, as well as performing (culturally specific Chinese) home-based exercises. Now he feels comfortable with fitting these exercises into his daily life. (C7, Mfinal)*
**Acceptability**				
App	User-friendly, despite initial challenges	*It's easy to use the app now. There were some modules that I didn't know how to add or use at the beginning. Now I know them, and I can basically do all kinds of operations, which is good. (P13, M3)*	Attractive	*I think the app is attractive, otherwise, the participants would not like to open the app. Now they usually log into the app. They feel nice with a coach guiding them through the tasks or processes, given they were quite friendly to me during the chat process. (C6, M3)*
		*The voice messaging function is good. If I text, I sometimes forget how to write. I'm 61 y old and I have not written for a long time. (P8, M3)*		*It’s sufficiently attractive. Because the education materials and its layout in the app is relatively novel. (C11, M3)*
Remote coaching	Convenient but, to some degree, not direct/personalised	*When I go to the hospital, the doctors are too busy to talk with me about health knowledge in detail. The advantage of the project is that we can chat [with the coach] about these things online. It’s very convenient. (P11, M3)*	Convenient, but sometimes delayed responses	*I reckon remote coaching itself is a trend of development and is also effective and practical because we cannot achieve large-scale in-person guidance, which is too costly. I think remote guidance is okay and easy. (C2, Mfinal)*
		*Regarding the way of leaving messages by the coach via the Chat function (of the app), I always feel like the leaving messages are from some Official Account (a verified account by a business or government in WeChat, capable of automatic messaging), thus feel impersonal … We should make an appointment by phone, or video call (before chatting via the app). (P1, Mfinal)*		*Regarding online communication, some participants are very active, they will take the initiative to send me some feedback, but some participants may ignore the messages, or they will reply to me a few days later. (C11, Mfinal)*
**Feasibility**				
Lifestyle change	Participant progress	*What kept me going? First, the visible weight loss was a big motivator. Second, my blood pressure became stable, and I could see real outcomes. Even though I still deal with occasional knee fatigue, my overall health has improved significantly. (P8, Mfinal)*	Participant progress	*If the participants improved their sleep or lost weight through exercise, or if their blood lipid levels decreased [improved], they would be more willing to continue participating. (C1, Mfina)*
	Partner support	*I believe we can encourage each other to exercise more. It's like, we could be each other's reminder, you know? (P7, M3)*	Partner support	*The couples participated together do better because they can encourage each other. (C9, 3M)*
		*Like greasy, spicy food, I also remind him to eat less. Sometimes he wants to eat fatty meat, but I don't make/cook it. (P6, Mfinal)*		
	Coach guidance	*I'm quite satisfied. To be honest, I probably couldn't stick with it if the coach didn’t regularly supervise me. (P13, M3)*	Coach guidance	*When they participated in this project, they felt like: I had a task, and my coach was helping me, so I had to do it well. (C4, Mfinal)*
	Reminders	*All kinds of reminders, slowly let us develop consciousness or habit. It's a little mandatory at first, a little boring, and you get used to it over time … Sometimes, I used to be too lazy to move, but now I have to exercise because I haven't finished my task / goal yet. (P5, Mfinal)*	Reminders	*Most participants still need reminders. Some of them may be too busy, so they didn't log into the app in time. When they were given a reminder, they logged in the app more frequently. (C5, M3)*
				*Some participants thought the app is beneficial because it can supervise them every day, they need to enter measurements and could get guidance from the coach, and then they paid more attention to improve blood pressure and blood glucose. (C5, Mfinal)*
Usage of app	Perceived smartphone literacy	*To be honest, I was poor at operating electronic devices at first … After setting my goals at the beginning (of the project), I learned how to enter the measurements, then I entered them every day. (P13, Mfinal)*	Perceived smartphone literacy	*For some elderly in China, although possessing a smartphone, they don't use it frequently and are not very good at using it. (C2, Mfinal)*
		*I'm just not used to using electronic devices … I don't know how to enter the measurements. It's more convenient for me to record the measurements in a notebook. (P1, M3)*		
	Available time	*For engaging over a longer period, I think there’s no problem because I don't have any tasks to do at home and thus have plenty of free time at my age. (P10, M3)*	NA	
		*Overall, I think this project is quite good, but I am not fully retired and still working, so my time available for health management and the app usage is still relatively limited because I rarely have a chance to rest when I am busy with my work / routines. (P13, M3)*		
Health coaching (using *Motivational Interviewing*)	NA		Lack of face-to-face contact is a barrier/Video contact is recommended	*I found the remote coaching/motivational interviewing a bit difficult because we can’t communicate instantly, and participants can’t give timely feedback. The speed of solving problem was too low. (C9, M3)*
				*Our hospital has an internet outpatient clinic. Based on this experience, I believe that if we could video participants for one or two minutes a day, the effect would be better. This would enhance communication as it feels more authentic. (C10, Mfinal)*
	NA		Coach meetings enhanced skills and self-confidence	*I felt I learned a lot from the coaching meetings. Because (coaches in) some of the centres in Beijing, did a really good job. I learned a lot about motivational interviewing. (C5, M3)*

C – coach, M3 – at approximately 3 mo into the PRODEMOS intervention, Mfinal – at 12 to 18 mo into the PRODEMOS intervention, NA – not applicable, P – participant, PRODEMOS – PRevention Of DEmentia using MObile phone applicationS

**Table 3:** Initial and sustained engagement and fidelity of the PRODEMOS intervention

	Intervention participants		Coaches	
	**Subthemes**	**Quotes**	**Subthemes**	**Quotes**
**Initial and sustained engagement**				
Participant - coach relationship	Trust built at baseline	*She gave very professional advice about weight loss when we first met …. She is cautious and patient, like a good friend … At first, my phone always failed to log into the app, but she patiently taught me how to do it on the spot every time. (P8, M3)*	Trust built at baseline	*The impression you leave on others in the first step (baseline visit) is crucial for whether they can continue to cooperate with you in the next step. For example, there was a participant who had difficulty in operating the app in the early stages, but she was still persevering, because she believed that I could help her. (C4, Mfinal)*
	Long-term relationship	*I think it's worthwhile to continue participating because the coach guides me and supervises me. It’s not that nobody cares about you after you participate. Instead, there is someone [the coach] in touch with you from time to time, advising you how to pay attention to your physical activity. I think it's good to be supervised, so I can keep going. (P13, M3)*	Long-term relationship	*When I followed up with participants, I began to get closer to them and understood their situation. Gradually, I felt that participants began to have a sense of trust in me, and then I felt that I was motivated, so I persevered to the health coaching. (C6, Mfinal)*
		*I’m very satisfied and the coach is strongly committed. The coach has given me empathetic guidance. I mainly wanted to lose weight, and she never tires of explaining and guiding me through the process. (C11, M3)*		*The participants came to thank us when they really changed their lifestyles, which is probably our biggest motivation for health coaching. I would feel that we play some role in their lifestyle change. (C3, M3)*
Influence of social environment	The role of the partner	*Sometimes when I read some tips (education materials) on topics like physical activity, blood pressure, and other health-related issues I think may be useful to us, I discuss it with my wife, and we can plan to do it together. (P5, Mfinal)*	Partner support	*The couples participated together do better because they can encourage each other. (C9, M3)*
		*She rarely eats meat and often advises me to eat less. However, she enjoys cooking meat dishes, and I can't control myself. The less she eats, the more meat I end up eating in a meal. (P11, M3)*		
	Social drinking and smoking, and holidays	*But when my diet is irregular, for instance, I eat out often during the Spring festival, my blood glucose tends to be high. (P2, M3)*	Social drinking and smoking, and holidays	*Some participants who want to quit smoking said: ‘I am sociable and don’t want to lose face. How can I refuse smoking if my friends offer me a cigarette when I meet them? I smoked their cigarettes, and I can't always take advantage of them. The next time we meet, I would offer cigarettes to them and smoke alongside them, which is a vicious circle.’ (C4, Mfinal)*
		*When I’m chatting or working with my colleagues if they offer me a cigarette, I also want to smoke, but I will try my best to restrain myself from smoking. (P7, Mfinal)*		
COVID-19 pandemic	Lockdown restrictions	*I used to work out in the gym, but it didn't last for long. Because of the COVID-19 pandemic, I was unable to go to the gym regularly this year, and the swimming pool was closed. (P14, Mfinal)*	Lockdown restrictions	*During the baseline visit, many participants were unable to attend the visit site due to the restrictions / lockdowns related to the COVID-19 pandemic. Consequently, we resorted to using video calls for communication. I believe communication via this way is not as effective as on-site visits, as it is easier to form friendship, become familiar with participants, and build trust relationships during on-site visits. (C5, Mfinal)*
		*But the year before last, I was not allowed to go out because of the epidemic of COVID-19, so I had to walk around the building, and then I started smoking again … I actually didn't feel very comfortable. I didn't want to smoke at that time, just because I couldn't go out. (P12, Mfinal)*		
**Fidelity**				
	Participants focused more on goals and education, less on chatting	*I did exactly as the goals that I’ve set. I was exhausted from walking one kilometer at the beginning. After gradually increasing the walking distances, I don't feel tired anymore to walk two kilometers … I couldn't have done those things all by myself (without support) … I often forget to enter measurements, but, overall, I still adhering to the goal. (P3, M3)*	Participants focused more on goals and education, less on chatting	*Yes, some of participants did forget to enter the measurements, but they are usually quite self-disciplined. For example, they went for a walk every day, and their diet became healthier. They silently implement it (the intervention) through their own way. (C1, Mfinal)*
		*I haven't chatted much with the coach (via the app). We've talked several times at the beginning about how to evaluate the goals. … When she (the coach) advised me on something, I just read the messages and followed the advice, but I didn't respond to her (the coach) because I didn't know/was unsure of what to say. (P2, Mfinal)*		*I think they read a lot of education materials. Usually, when I send it (from the coach portal) to the app, they will read it. But they don't like to chat with me, rarely reply to my messages, or simply answer 'OK'. (C6, Mfinal)*
				*Some participants were not accustomed to using the app (chat functionality) and preferred direct communication via WeChat (i.e. a popular Chinese instant messaging and social media app), as they were used to contacting others through it. (C10, M3)”*
	Using the platform except for technical issues or prolonged non-response	*The coach normally contacted me via the app … but when I couldn’t log in to the app occasionally, I sent her a message on WeChat, and she replied to me to guide me on how to operate it or called me. (P8, M3)*	Using the platform except for technical issues or prolonged non-response	*I usually told them how to use the app in the ‘Chat’ and sent them a screenshot that marked where to enter measurements and set goals. I would call them if they didn’t reply or understand. But sometimes it would be messy because they didn’t understand when I explained how to use the app over the phone. (C1, M3)*
				*We seldom communicate in other ways. I only called participants when they didn't fill out questionnaires on time or didn't reply to my messages for one or two weeks. (C11, M3)*

C – coach, M3 – at approximately 3 mo into the PRODEMOS intervention, Mfinal – at 12 to 18 mo into the PRODEMOS intervention, P – participant, PRODEMOS – PRevention Of DEmentia using MObile phone applicationS

#### Appropriateness

App/coach platform. Both participants and coaches perceived the PRODEMOS platform as instrumental in providing supervision and guidance. The educational materials and coach’s dedication and direction were reported as pivotal in enhancing health awareness among participants and meeting their needs for reliable health information, particularly considering the varied and inconsistent quality of online health information. Coaches emphasised the significant support provided by the educational materials on the coaching platform. Goal-setting proved beneficial for behavioural change, as participants expressed a willingness to exert greater effort towards achieving their goals once set one.

Health coaching. Participants indicated that the coaching they received was detailed and precise. They particularly appreciated guidance on dietary adjustments and balanced physical activity. This tailored approach not only addressed their individual needs but also significantly facilitated the practical application of behaviour modification strategies. Coaches recognised that MI could enhance participants’ intrinsic motivation. Additionally, customised guidance was deemed crucial in participant-coach communication, with participants being more responsive to coaches perceived as friends rather than as patronising or authoritative figures.

Lifestyle change. Some participants indicated that goal-setting and/or coaching strategies facilitated the integration of behaviour change into their daily routines, such as combining caregiving responsibilities with increased exercise commitment, making it become habitual.

#### Acceptability

App/coach platform. Participants generally found the app easy to use, despite initial challenges during the early phase. The voice chat functionality was preferred by those with typing difficulties, although some expressed concerns about potentially disturbing their coach or encountering misunderstandings due to their coach’s potential difficulty in fully understanding their speech because of local dialects or accents. Overall, the app was deemed acceptable, useful, and easy to use. The coaching platform notably provided support for coaching, particularly for actively involved participants.

Remote coaching. Both participants and coaches felt remote coaching was agreeable, although its effectiveness was somewhat reduced for participants who logged in the app less frequently, resulting in delayed feedback. One participant, dissatisfied with the messaging approach, expressed a preference for more direct communication like video or phone calls.

#### Feasibility

Lifestyle change. Participants and coaches agreed that various factors strongly influenced lifestyle changes in this study. These included participant progress, partner support, coach guidance, and the app's reminders.

This study highlights the pivotal role of goal achievement in driving subsequent behaviour change among participants. Achieving specific goals or making significant progress boosted their sense of accomplishment and confidence, driving deeper engagement in the behaviour change process. In the one partnership represented, both noted that mutual reminders and exercise support facilitated behaviour changes. Automated or coach-initiated reminders instilled a sense of responsibility in participants, aiding them in overcoming inertia. Attentive supervision ensured adherence to the intervention, with participants more likely to stay committed when they felt connected to and supported by the coach.

Usage of the app. Technical difficulties with the app, such as login issues, discouraged usage. Login issues, primarily due to older participants forgetting the login procedure explained during the baseline visit, were reported by a small percentage of participants ( ~ 5%). While these issues did not substantially affect overall engagement, they contributed to occasional delays in app access and communicating with coaches. However, participants who were eager to learn, especially after overcoming specific challenges and experiencing benefits, tended to explore its functionalities more thoroughly, reinforcing engagement. In terms of commitment, most participants, especially those who were retired, did not perceive long-term engagement as burdensome. They viewed it as a valuable and engaging activity. However, one participant, who was not retired and had a busy schedule, consistently struggled to find time for app usage, ultimately leading to discontinuation from the study.

Health coaching (using MI). In general, remote health coaching via the app was feasible; however, communication efficiency was hindered by slow feedback. The lack of instant visual cues, as seen in face-to-face or screen interactions, caused delays in coach-participant responses and made conducting MI challenging.

Additionally, monthly group interactions among coaches were considered a key facilitator in enhancing MI skills and expertise.

#### Initial and sustained engagement

Participant – coach relationship. The authority of recruitment through well regarded hospitals and the initial face-to-face guidance from a coach during the baseline visit established a foundation for participant engagement. In this context, trust between participants and their coach played a crucial role in fostering engagement. Furthermore, both participants and coaches perceived that a strong, long-term relationship between participant and coach contributed significantly to sustained participation in the study. The cultivation of positive rapport emerged as a fundamental element influencing continued involvement. Participants consistently expressed deep appreciation and trust in their coaches during interviews, attributing their sustained engagement. This trust played a crucial role in motivating participants to invest more effort in lifestyle changes, akin to the support received from a trusted friend. For coaches, the trust placed in them by participants further served as an impetus for their continued dedication.

Influence of social environment. Long-term engagement can be enhanced by partners participating in activities together, such as exercising or cooking. While this is considered a facilitator to lifestyle change, it may also act as a barrier to dietary modifications due to the varying cooking and dietary preferences of the partner.

In the context of social interactions, various temptations and prevailing social norms significantly impacted participants' attempts to change lifestyle behaviours. One participant struggled to quit smoking, citing the challenge of resisting cigarettes offered by colleagues or friends at social gatherings. Additionally, some participants indicated irregular diets during holidays, like the Chinese Spring Festival, characterised by a tendency to consume rich and greasy foods while entertaining guests.

COVID-19 pandemic. During the COVID-19 pandemic, lockdowns and restrictions impacted participant engagement. Some baseline visits had to be conducted online, in addition to participant reluctance to attend in person. Remote explanations of the app and building trust were challenging. Exercise levels varied, with some reducing outdoor activities while others transitioned to indoor exercises. Participants reported experiencing low moods and increased smoking, attributing these changes to reduced social interactions and outdoor activities.

#### Fidelity

Most participants adhered to their set goals, although some logged in less frequently or entered measurements less often, mainly due to perceived inconvenience and forgetfulness. Many implemented lifestyle changes based on coaching and educational materials; however, they also noted they often neglected to update their goals in the app.

In the early phase, participants communicated more frequently with their coaches, primarily for technical support. Although coaches initiated most conversations, some participants typically followed their guidance and reminders without extensive dialogue. Some participants noted they initially engaged with the app out of curiosity and the need for practice with technology, but then found they transitioned to adopting healthier lifestyles as they gained proficiency with the app. For some, setting goals and entering measurements supported behaviour change, while a few found the app became less appealing over time.

Participants mainly focused on their goals and educational materials, communicating less with coaches and often acting on advice directly. Interaction occurred primarily through the platform, except in cases of technical issues or prolonged lack of response. Chatting via messaging was relatively challenging for both participants and coaches. Some participants found it difficult to chat through a new, previously unknown app, while coaches struggled to pursue MI remotely in an efficient manner, lacking direct face-to-face contact.

### Recommendations for future improvements of the app and coaching

Both participants and coaches suggested the app’s visual appeal could be enhanced by incorporating video-based educational materials, including Traditional Chinese Medicine (TCM) practices like Chinese traditional acupoint massage or standing meditation. Additionally, there was a consensus on the importance of integrating voice and/or video call features to improve communication within the app.

*The app should be designed to be more concise, with fewer entering procedures (should minimise the need for entering measurements), and a more cartoonish style similar to WeChat (a popular Chinese instant messaging and social media app) Movement, which is one of WeChat's functions and only involves answering some multiple-choice questions instead of entering something (measurements)*. – P1, M3

*They (participants) need something that can guide them in daily practice…. For example, what and how much you should eat. I think if we make this into a mini video advising participants how to do it, then they can visually assess portion sizes of the food, so that they will be very happy and know how to eat healthier.* – C5, Mfinal

Participants suggested conducting additional follow-up visits with physical examinations and personalised consultations, supplementing existing health care services. They also expressed anticipation for the development of a mobile app for coaching to replace the current computer-based coaching, making coaching more flexible and convenient, thus enhancing overall efficiency.

*I hope the coach can communicate with the participants via either video call or meeting in person once a month or every two months. If we can consult with our coach or doctor when we have some minor health problems, we will feel particularly good and more secure. –* P11, Mfinal

*If the coach had a work platform that could communicate with participants through mobile phones, like through WeChat, it would be relatively more convenient for instant messaging. –* C10, Mfinal

Participants and coaches recognised the PRODEMOS intervention approach as a convenient supplement to health care. They reported it can provide guidance for managing health via smartphones, reducing the need for frequent medical visits. They raised the potential to be beneficial for health care provision from a broader perspective provided it is integrated into primary care.

*When I go to the hospital, it takes a lot of time to talk about my condition in detail, and the doctors are very busy. The advantage of the project is that we can communicate about these things online… and the app and coach always give me advice that I can follow. It would be particularly good if the health system does as this project, particularly in community hospitals*. – P11, M3

## DISCUSSION

### Summary of main findings

Our findings indicate that the Chinese participants viewed the PRODEMOS intervention as an appropriate and acceptable method for modifying their lifestyle behaviours to reduce dementia risk factors. This perception was influenced by the effectiveness of the app and health coaching in improving health awareness and meeting individual needs. Coaches highlighted the importance of MI in strengthening participants' intrinsic motivation. However, they encountered challenges in effectively applying MI techniques due to the lack of face-to-face or video communication and delayed participant responses, which impeded communication efficiency. The feasibility of implementing the intervention hinged on several critical factors, such as its integration into daily routines, participant progress, partner support, the level of attention and supervision provided by coaches, perceived smartphone literacy, and time availability. Both initial and sustained engagement with the intervention were influenced by various factors, including the participant-coach relationship, the surrounding social environment, and the impact of COVID-19 pandemic. Conversely, the extent of coaches’ involvement depended on participant responses and progress. While participants generally adhered to goals, fidelity varied due to challenges such as balancing caregiving responsibilities, managing stress, encountering technical issues with the app, and differing personal preferences. These factors affected the regular input of measurements, lifestyle change, and communication with the coach. Notably, both participants and coaches endorsed the future integration of the PRODEMOS intervention into primary care settings, highlighting its potential for large-scale and sustainable implementation.

### Comparison with existing literature

Prior to this study, most mHealth research on Chinese populations focused on managing cardiovascular diseases, obesity, and diabetes, or individual risk factors like physical inactivity. However, these studies lacked professional human support and did not address dementia prevention – a gap our study aimed to fill [11,15]. Additionally, our results align with international research showing that motivation to participate was driven by expected health benefits and altruism [21,22], with a unique motivator among Chinese participants being the desire to avoid becoming a burden to their children. A recent small-scale randomised controlled trial (n = 48) observed that a six-month multidomain intervention – delivered via WeChat messages on dementia literacy and behavioural guidance – may improve dementia risk profiles and cognitive performance in at-risk older adults in Northeast China. The study suggests that WeChat-based interventions, combined with support from health care workers and family, can be effective in reducing dementia risk among Chinese older adults, aligning with our findings [23].

Regarding appropriateness, participants perceived the intervention as relevant to their lives. Key factors included perceived benefits, participant-coach relationships, partner support, and integration into daily routines, which were important for engagement. The use of MI techniques at the study baseline enhanced intrinsic motivation and supported self-management, facilitating behavioural changes that fit smoothly into daily life, consistent with findings from European studies [7,10,24]. Additionally, the availability of reliable educational materials ensured participants had access to authoritative information, essential for informed decision-making.

In terms of acceptability, our study revealed that Chinese participants valued the user-friendliness of the PRODEMOS app and prioritised access to reliable health information. Those with limited medical knowledge faced challenges in identifying credible online health resources – a difficulty noted in previous research [25]. Coaches observed that the rigorous review process for educational materials further enhanced acceptability by providing participants with trustworthy resources. This finding aligns with research from England on a digital psychoeducation intervention, where combining diverse knowledge sources with remote, flexible, and personalised support significantly improved acceptability [26].

Feasibility of engaging participants and providing effective remote coaching was supported by findings that align with previous research on digital interventions for healthy ageing and cognitive health. From the participant perspective, challenges such as limited smartphone literacy and low self-confidence among older adults were evident, particularly in China, where educational backgrounds often differ from those in Western countries [27,28]. Participants expressed a preference for simple, visually engaging health materials, such as animations and videos, reflecting findings from research on digital device literacy [28]. Support from both coaches and partners proved crucial for sustained engagement, as shown in the HATICE study [10], helping participants address technical difficulties, maintain motivation, and adapt to lifestyle changes [29,30]. However, partner involvement was not always beneficial; differences in cooking preferences sometimes created barriers to dietary adjustments, a challenge echoed in other culturally diverse settings [31].

From the coach perspective, the feasibility of remote support was impacted by the asynchronous nature of app-based communication, which often delayed feedback compared to in-person or video sessions. To address this, coaches scheduled regular check-ins via messaging or phone calls, providing reliable support touchpoints. They encouraged participants to send updates at their convenience, committing to timely responses to maintain feedback continuity. Coaches also supported technical feasibility by providing user-friendly guides and video tutorials, empowering participants to troubleshoot independently and maintain app engagement. Personalisation was emphasised, with goals tailored to daily routines, such as combining physical activity with caregiving tasks. Coaches adapted motivational techniques based on feedback, offering specific exercise suggestions and celebrating even small achievements through personalised messages. While this approach helped sustain motivation, variability in adherence remained, underscoring the need for consistent engagement strategies – such as coach reminders and fresh content, as seen in studies like the UK’s digital diabetes prevention programme and a UK weight loss trial [32].

Adoption is essential for sustained engagement, and while some participants initially faced technical challenges, consistent coach support played a pivotal role in getting them started – a finding that aligns with studies using digital tools for chronic kidney disease self-management [33]. The simplicity of the app’s interface was widely appreciated, especially by those with higher smartphone literacy, who reported stronger initial engagement [14]. However, sustaining this engagement is often challenging; a prior systematic review and meta-analysis indicated that the effectiveness of digital health interventions tends to diminish, particularly beyond 12 months [34]. This aligns with participants’ feedback in the second round of interviews, where a sense of monotony with the app’s content emerged, coupled with requests for more diverse health education and broader goal-setting options.

Trust and personalisation also proved fundamental for long-term engagement. Initial face-to-face contact helped establish a trusted participant-coach relationship, which motivated sustained involvement [10]. For example, coaches observed that voice chats and video calls were especially effective for participants with limited digital literacy, as these formats felt more personal and convenient than text messaging. This preference highlights the need to maintain flexibility in communication styles to strengthen the emotional bond, especially as simply exchanging text messages was often insufficient for building the deeper connection required for trust. Additionally, some participants preferred goal-setting and educational materials over direct social support from the coach, possibly reflecting a cultural tendency among Chinese individuals to prioritise professional advice as well as the impacts of social isolation during the COVID-19 pandemic.

Social influences also shaped sustained engagement. Supportive partners who participated in shared activities like exercising or cooking encouraged lifestyle changes, yet differing dietary preferences sometimes presented barriers. Furthermore, social situations, such as gatherings where participants encountered smoking or festive, calorie-rich foods, posed challenges to maintaining behavioural changes. This points to the importance of incorporating strategies to help participants navigate social temptations and norms that can hinder sustained engagement with health goals.

### The influence of COVID-19

The impact of COVID-19 on the PRODEMOS intervention appears to have been bidirectional. Survey data shows that mHealth app usage nearly doubled during the pandemic, driven by heightened health awareness and the need to limit in-person consultations [35]. However, lockdowns also led to decreased physical activity among older adults due to restricted access to exercise facilities [36]. Changes in daily routines and limited social interaction also affected motivation and adherence. In response, the intervention was adapted to provide increased remote support from coaches and set goals tailored to participants' changing circumstances. Some participants faced setbacks, such as relapses into smoking or reduced physical activity, which required additional motivation and tailored guidance from coaches. Conversely, other participants reported minimal impact on their physical activity and credited their coach's recommendations for indoor exercises, such as practicing traditional Chinese exercises like Baduanjin Qigong and standing meditation at home. Regarding the PRODEMOS study itself, the COVID-19 pandemic affected the logistics and support of the study intervention, as some participants faced challenges attending baseline visits or resolving technical issues on-site, such as login problems.

### Strengths

An important strength of this study is its qualitative interviews at two time points to assess the experiences and views of both participants and coaches regarding short- and long-term implementation outcomes of the mHealth intervention. Conducting interviews with both participants and coaches facilitated mutual validation and supplementation of implementation outcomes through triangulation, enabling a comprehensive analysis of changes in engagement, variations in motivations, and underlying influencing factors. The fact that all eleven participating coaches were willing to join the interviews, including the one who had previously stopped coaching for the study, optimises the evaluation of their views and experiences. Finally, another potential strength lies in having one interviewer (JZ) present throughout all interviews, ensuring consistency in interview guidance and analysis.

### Limitations

Some interviews were conducted via video conferencing on WeChat due to COVID-19 pandemic restrictions. Although this approach may have limited non-verbal communication, we believe its impact was minimal, as our conversations with participants were open and frank. We do not anticipate selection bias resulting from video call interviews, as participants received assistance from family members with technical issues, and none declined interviews due to difficulties with video calls. Nevertheless, despite purposive sampling at the start of the study, there may be selection bias toward positive perspectives, as PRODEMOS participants were likely more motivated and digitally skilled. Thus, generalisation to the broader eligible population should be approached with caution. However, since all 14 participants also took part in a second interview (including one participant who had dropped out), there was no further selection occurring during this study.

### Implications for practice, policy, and future research

The PRODEMOS app emphasises the importance of personalised goal-setting in digital health interventions by adapting features to individual preferences and cultural traditions, consistent with findings from previous studies [10]. Participants expressed a desire for more personalised options, especially incorporating culturally specific Chinese exercise traditions such as Baduanjin Qigong and TCM practices. Many also preferred voice or video communication with coaches to enhance trust and engagement. These integrated features could support behavioural change and improve communication quality, facilitating stronger relationships between coaches and participants. Additionally, both participants and coaches recognised the potential for integrating the app with the health care system to enhance support and improve compatibility with health monitoring devices.

The implementation of the PRODEMOS intervention in a middle-income country illustrates the feasibility of integrating mHealth solutions into public health strategies for dementia prevention. Policymakers should prioritise linking mHealth tools with Chinese community hospitals and organising health education events, particularly in smaller cities and remote areas. Creating a unified platform that consolidates various health management apps could enhance preventive care and streamline patient support. Collaboration with decision-makers and prevention experts is essential for the sustainable adoption of these tools within existing health care infrastructures, which could ultimately improve long-term engagement and adherence.

Future research should focus on refining motivational interviewing (MI) techniques and evaluating how different communication styles, such as voice and video calls, affect participant engagement compared to standard text messaging. Additionally, investigations into the long-term sustainability and scalability of the PRODEMOS intervention in various health care settings are needed. Research should also address factors influencing participant adherence, including technical challenges and coaching styles, while assessing the impact of consistent support, tailored goal-setting, and regular reminders on motivation and engagement beyond the 12–18-month intervention period.

## CONCLUSIONS

This study, the first qualitative evaluation of the Chinese arm of the PRODEMOS intervention – an interactive, coach-supported mHealth app to reduce dementia risk factors among older adults – assessed experiences and views of participants and health coaches regarding its implementation. The intervention was found to be acceptable and implementable for promoting lifestyle changes in individuals at increased dementia risk. Despite challenges in remote coaching and variability in participants’ adherence, positive feedback highlights its potential for integration into primary care and large-scale implementation, provided issues with coaching and sustained engagement are addressed. These findings offer valuable insights for practitioners and policymakers seeking to integrate mHealth solutions into public health strategies for dementia prevention.

## Additional material


Online Supplementary Document
