# Supplementary material for: Implementation of a coach-supported mHealth intervention for dementia prevention in China: a qualitative study among Chinese participants and coaches in the PRODEMOS trial
Source: J Glob Health. 2025 Mar 28;15:04036. doi: 10.7189/jogh.15.04036 (PMC11950902; doi:10.7189/jogh.15.04036)
Supplement: Online Supplementary Document [file jogh-15-04036-s001.pdf]

Supplement to: Zhang J, Hoevenaar-Blom MP, Jian X, Hou H, Ge S, Brayne C, Eggink E, Hafdi M, He M, Wang G, Wang W, Zhang W, Yu Y, Niu Y, Lyu J, Song L, Wang W, Wang Y, Moll van Charante EP, Song M; PRODEMOS study group. Implementation of a coach-supported mHealth intervention for dementia prevention in China: a qualitative study among Chinese participants and coaches in the PRODEMOS trial. J Glob Health. 2025;15:04036.

Appendix S1. Topic list for the intervention participants and coaches of PRODEMOS in China after three months of intervention

1.1. Topic list for the intervention participants of PRODEMOS in China after three months of intervention

-Overall evaluation of intervention (as a whole) (**feasibility**)
-Overall evaluation of application
-Overall evaluation of coaching

<u>Specific implementation outcomes</u>

Introduction

-What made you decide to participate? (Is the decision to participate made by yourself or by your family/friends? Did you take the initiative to participate after seeing the recruitment advertisement, or you were invited to participate by a practice person of the study?)
-Did you change your lifestyle due to the intervention? E.g. join a sports club? If not, what were the barriers? (if the intention was to change lifestyle)

Adoption

-Did you set a goal/goals at the start?
    -Which goal(s)
    -Why those goal(s)
    - Did the app sufficiently facilitate you to set the goals as you wished to do? (in terms of type of goal, how to achieve it, how to measure it, etc.)
-Did you enter any measurements? (for what?)
-Did you communicate with the coach through the chat? (if not, otherwise?) How did this go? Was it working well?
- What did you do when your goal reached its evaluation date? Did you use the goal evaluation section?
- When you need(ed) medication support, did (do) you go to the hospital or adjust medication by yourself? Do you think the app or coaching needs to add medication support section that tailored to your needs in the real-life intervention?
-Did you use the advice and education section?
-What do you think of the advice or explanations in the education section of the app? Are

they useful/credible? Did you miss specific things?
-Did you visit your GP or other healthcare professionals due to the intervention? E.g. in relation to lifestyle, high blood pressure or high cholesterol?

Appropriateness
-Does the intervention fit your needs to make your lifestyle healthier?
-Does the intervention fit well enough in your life?

-Is the combination of app use and coaching useful for you? Or would one or the other be sufficient?
-Did you communicate with the coach outside the app, e.g. via phone calls? If not, did you want to have the option to call your coach?
-What aspect(s) do you think the app needs to be improved at (Layout, goal category, educational materials, etc.)? What aspect(s) do you think need to be improved regarding the coaching?
- Do you think the intervention is an addition to regular healthcare?

Acceptability
-Do you find the intervention as a whole appealing?
-Do you find the application sufficiently user-friendly?
-Is it enjoyable/agreeable to participate in the study/to use the app?
- Do you like working with the levels/ badges?
-How do you rate the advice or suggestions made by the coach?
-Do you find the advice or explanations in the education section of the app useful/credible?
-Is the overall use of the app acceptable? (easy or (too) complex?)

Sustainability
-Was it easy/acceptable/difficult to engage with the intervention over a longer period of time?
- Do you need to have face-to-face contact/consult with your coach every now and then (e.g. every month, quarter, or year) to support you to engage with the intervention/to change your lifestyles over a longer period of time or in real life?
- Can you describe whether you used the app more intensive in certain periods of the trial?
-What kept you going? Reminders? Contact by the coach? Other factors?
-Did you consider quitting? (why?)
-Did you set a new goal during the study? If so, was this prompted by a suggestion from the coach/ If so, how did that come about? If not, what barriers did you encounter? [pre-structured]
-Idem, for measurements, and use of chat function or education section
-What was the role of your peers/partner in the intervention? Did they stimulate you to keep going with the intervention?

Feasibility
-Were there any practical barriers to use the app? For example, problems with logging in, or not getting reminders? If so, which?
    E.g. technical issues (login/chat/no reminders)
-Were there any social barriers to use the app? If so, which?
    E.g. bad contact with coach, education material not appropriate
-Were there any practical barriers to change your lifestyle? If so, which? E.g. gyms were closed due to COVID regulations?
-Were there any social barriers to change your lifestyle? If so, which?

Fidelity

-Compared to your expectations at the beginning of the study, did things work out as you expected in terms of achieving some of your goals?
- Do you know the rationale/concept of preventing dementia in PRODEMS？Did your coach well explained to you that the prevention of dementia of this study would be achieved through targeting cardiovascular risk factors instead of brain training (e.g. puzzle games)? If you had expected the intervention through brain training, instead of/on top of targeting cardiovascular risk factors, how did your coach explain to you?
- Did your lifestyle change due to PRODEMOS? If so, in what way?
- Did the PRODEMOS intervention help you obtain/achieve their goals?

1.2 Topic list for the coaches of PRODEMOS in China after three months of intervention

General views and experiences
-Overall evaluation of intervention (as a whole) (**feasibility**)
-Overall evaluation of application (Coach Portal and perception of participant use)
-Overall evaluation of coaching

Specific implementation outcomes

Introduction
-Expectations about intervention and/or coaching

Appropriateness
-Does it fit the needs of the target population?
-Can participants sufficiently use this app, or will they need your help throughout the study?
-Focus on self-management or coaching?

Acceptability
-Do you think the intervention as a whole is sufficiently attractive and user-friendly for participants?
- Did you well explain to participants that the prevention of dementia will be achieved through targeting cardiovascular risk factors instead of brain training, at the start? Could participants understand and accept the rationales of preventing dementia in PRODEMS?
-Is it enjoyable for participants to use the app?
- Do you think using the app is too complex for some participants?
-Do you think educational level appears related to health awareness and use of the intervention?
-How did you like the training before starting? Did it give you sufficient confidence you could perform this task yourself?
-Does the platform sufficiently help you to support the participants? If not, how do you achieve/go about this?
-What aspect(s) do you think the platform (i.e., the app and the Coach Portal) needs to be improved at?

- Is it easy to remotely coach participants?
-Do you find the advice or explanations in the education section of the app useful/credible to use in your coaching?
- Do you think the intervention (app and coach) is an addition to usual healthcare?

Adoption
- Was the coach protocol sufficiently clear, detailed and operationalised for use in daily practice
-Were there aspects of the protocol that did not work for you? How did you handle this?
-To what extent you use the Coach Portal? (Which functionalities? How often?)
-Which features were useful, and which ones were not?
-How did you use the Coach Portal in your coaching?
- Did you communicate with participants outside the portal, e.g., through a phone call? If so, what was the reason? And how often did you do this?

Sustainability
-Was it easy/acceptable/difficult to engage with the intervention over a longer period of time?
-Which app functions were used well or less well (and why)?
-What kept you motivated to continue coaching? Facilitating or impeding factors for yourself? And for participants? What kept them going, what were barriers/facilitators?
-- When participants thought that changing their lifestyle was a gradual/lengthy process, and thus they expected immediate treatment, how did you handle this, to still convince the participants to work on their lifestyles?

Fidelity
-Did you manage applying the motivational interviewing techniques? Can you give an example?
- How did you handle it when a participant needs to consult for his/her medication use?
- What are the reasons why participants are not active? How did you handle it?
- How did you handle it when participants think they are too busy with their daily routines or works to have time to do regular physical activity or exercise? What kind of ideas did you have in mind to strengthen the coaching in the future when participants do not adhere to the intervention because of their busy work or family obligations?
- When participants thought that changing their lifestyle was a gradual/lengthy process, and thus they expected immediate treatment, how did you handle this, to still convince the participants to work on their lifestyles?
-How did you like the scheduled meetings to discuss problems and the progress of your coaching?
- Do you think the Coach Portal sufficiently facilitated you in the coaching of the participants toward their goals?
- Which parts of the coach platform did you use most? And which did you not use?
- To which degree did you aid the participants in goal setting/changing/attaining over time, and provided advice through the chat function over the study period?

Appendix S2. Topic list for the intervention participants and coaches of PRODEMOS in China at final assessment

2.1 Topic list for the intervention participants of PRODEMOS in China at final assessment

Introduction

- Now that the study is coming to an end: what is your opinion about the study as a whole? What do you think were the strengths and what could be improved?
- What goals did you have at the beginning of the study?
    - To what extent did you achieve those goals?

- Can you explain how you used the app in your daily/weekly routine?
  -Did you find the study as a whole appealing?
  -Did the study fit your needs to work on a healthier lifestyle?

-Did you find the study fit well enough in your life?
  If yes: can you explain how the intervention fits into your daily/weekly routine?
  If not: why not? Is there something that could be improved to help with this?

App

-What is your opinion of the app? What are the strengths and what can be improved?

- Did you find there were times that you used the app more frequently or less frequently?
  If yes, what prompted your changes in use?

-Did you set any new goals throughout the study?
  If so, was this prompted by a suggestion from the coach? If not, what made you chose to set a new goal? OR: What is the reason you did not set a new goal?

--Do the types of goals need to be increased or changed?
  If so, do you think the goals related to health promotion with Traditional Chinese Medicine approach (e.g., massage acupoints) need to be added in the App?

-Did you enter any measurements throughout the study? (for what?)
  If so, was this prompted by a suggestion from the coach? If not, why did you choose not to enter measurements? Was it useful for you to have an overview of your measurements/to monitor your measurements?

- Did you use the library throughout the study? Did you use it mainly in the beginning or also later in the study?
- Did you read the monthly news items that appeared on your to-do list?
  If so, what did you think of them? If not, why not?
- China only: Did you use the voice messaging? Why (not)? Do you prefer this over text messaging? Why (not)?

Coaching

-Did you use the chat messaging to contact your coach throughout the study? How did you find this?

- Did you find that you talked to your coach the same amount throughout the study? Or was there a period that you communicated more, and how was this initiated?

Was there a period you did not communicate with your coach? If so, what was the reason?

-Did you communicate with the coach outside of the app, e.g. via phone calls?

If not, did you want to have the option to call your coach? With or without video? Or voice messaging?

-What is your opinion of the coaching?

-Did the suggestions made by your coach meet your needs?

If yes, in what way; can you give an example? If not, how could that be improved?

-Is the combination of app use and coaching useful for you? Or would one or the other be sufficient?

<u>Intervention</u>

-How did you find participating in the study over a longer period of time?

If not discussed previously: Did you participate to the same extent throughout the whole study? Or were there periods you made use of it more or less? How did this come about?

-What kept you going?

Reminders? Contact by the coach? Success in reaching a goal? Other factors?

-Did you consider quitting? (why?) Did you pause the study? (why?)

-Did you have any support from friends or family?

If yes, in what way? Did they encourage you to continue with the study?

-Did you change your lifestyle due to the study?

If so, in what way? Do you think you will continue with the changes you have made?

If not, what were the barriers to you making changes?

-Were there any practical barriers to change your lifestyle?

If so, which? E.g. gyms were closed due to COVID regulations?

-Were there any situations where it was difficult to change your lifestyle?

E.g. in social venues, or no support from friends/family. If so, which?

- Do you intend to continue working on your lifestyle now that the intervention is (almost) finished?

If so, how?

- And hypothetically: would you have liked to continue using the PRODEMOS app or would you use another lifestyle app or none at all?
- If similar apps were launched within the NHS [or Chinese equivalent], would you participate again?
- what role do you think GPs should play in coaching patients?
- Do you have other tips for us for future interventions?

2.2 Topic list for the coaches of PRODEMOS in China at final assessment

<u>Intervention as a whole</u>

- What is your opinion about the intervention as a whole? What are the strengths and what can be improved?
- How was it to engage with the intervention over a longer period of time?
- What kept you motivated to continue coaching? Facilitating or impeding factors for yourself?
- What do you think of the engagement of participants throughout these 12-18 months?
- What kept them going, what were barriers/facilitators?
- What is your idea on the characteristics of participants that stop/quit/do not engage with the study? And which participants are more likely to engage well?
    - Do you have suggestions on how to improve the engagement of participants?

App

- Which app functions were used well or not well over time (and why)?

Were there parts of the app that participants kept using for the whole 12-18 months?

Were there parts of the app that participants did use very little or not at all during the study?

Coaching

- Can you tell me about your experiences on coaching people over a longer period of time, that is: beyond the first couple of months, over a period of up to one-and-a-half year?
- Can you give me examples of participants that appeared successful? How did you (try to) coach them and what have you learned to improve your coaching?
- Did you manage applying the motivational interviewing techniques? Can you give an example?
    - What is your experience with the motivational interviewing techniques? Were you familiar with these techniques prior to PRODEMOS? Do you think it helped you to coach participants?
- Can you give examples on techniques in coaching that worked well and techniques that did not work well?

- What is your opinion about coaching remotely for 12-18 months? Is it easy/difficult? Why?
- Can you give examples of participants for whom the remote PRODEMOS intervention worked (fairly) well and of participants who needed more personal forms of contact, including phone calls and/or F2F contacts? [In what ways did they differ, in terms of personality, type of goal, or otherwise?]

Coach Portal

- What did you think of the Coach Portal?
    - Did you communicate with participants outside the portal? E.g. via email or with a phone call. If so, what for?
    - Did you use spreadsheets outside the portal to keep track of participants? If so, for which purpose? Did you miss anything in the Coach Portal?

- Did you find the advice or explanations in the education section of the app useful/credible to use in your coaching? Do you miss certain topics? Could you also use it later in the process to keep people interested?

Coach protocol

- What did you think of the coach protocol?
- Did you use the protocol throughout the study? If so, in what way? If not, why not?
  In the past months, how much did you look at it?
- Were there aspects of the protocol that did not work for you? How did you handle this?

Coach meetings

- How did you like the scheduled meetings to discuss problems and the progress of your coaching? Did this change over the course of the study?
  - Which aspects of these meetings were valuable for you? (any examples?) Would you have any suggestions to improve them? (i.e. would it help if people with certain expertise (in MI, coaching, etc.) would attend such meetings?)

Future use

We would like to improve the intervention for future use.

What advice would you give us on adaptations or redesign?
Should it be offered as part of health care prevention in primary care?
What is needed to implement the intervention in regular healthcare?

Table S1. Characteristics of the intervention participants (n=14)

**Participant characteristics**										**Study characteristics**			
**No.**	**Age (years)**	**Gender**	**Living situation**	**Education level***	**Retired**	**DM**	**CVD**	**No. of risk factors†**	**Region**	**First FU duration (mo)**	**Second FU duration (mo)**	**Partner participating**	**Login frequency (per mo)**
**P1**	71	M	Spouse	1	Y	Y	Y	3	Beijing	3.1	13.5	N	53.5
**P2**	68	F	Child; grandson	3	Y	Y	N	4	Beijing	3.0	13.5	N	19.3
**P3**	68	F	Spouse	4	Y	N	N	2	Beijing	2.7	12.5	N	30.1
**P4**	61	M	Spouse; children	4	Y	N	N	3	Beijing	2.8	14.2	N	26.5
**P5**	70	M	Spouse; children	4	Y	N	Y	3	Beijing	3.0	16.1	Y	54.9
**P6**	56	F	Spouse	2	N	N	N	4	Tai'an§	3.1	13·2	Y	12.0
**P7**	56	M	Spouse	4	N	N	N	6	Tai'an§	2.6	13.2	Y	2.9
**P8**	60	F	Spouse; children	2	Y	N	Y	5	Beijing	2.8	12.5	N	78.1
**P9**	69	M	Spouse; children	2	Y	N	Y	4	Beijing	3.1	12.5	N	16.5
**P10**	58	F	Spouse	2	Y	N	N	4	Beijing	3.0	12.1	N	34.5
**P11**	58	F	Spouse	3	N	N	N	4	Beijing	3.1	12.3	N	12.6
**P12**	71	M	Spouse	2	Y	N	Y	3	Tai'an§	2.7	12.5	N	20.1
**P13‡**	58	F	Spouse	4	N	N	N	3	Beijing	2.6	14.6	N	9.2
**P14**	57	M	Spouse	4	N	N	N	4	Beijing	2.8	12.1	N	19.6

CVD – cardiovascular disease, DM – diabetes mellitus, F – female, M – male, Y– yes, N – no, FU – follow-up, PRODEMOS – PRevention Of DEmentia using MObile phone applicationS, mo – month.

*1 – primary school and below (ISCED level of 1), 2 – Junior high school (ISCED level of 2), 3 – Senior high school (ISCED level of 3), 4 – College and above (ISCED level of 4).

†Risk factors include physical inactivity, active smoking, hypertension, cardiovascular disease, diabetes mellitus, dyslipidemia, obesity, and depression.

§A prefectural/small city in Shandong province, China, with 5.5 million inhabitants.

‡The participant no. 13 (P13) attended the follow-up interview at the end of the trial, although she quit the PRODEMOS trial at seven months into the intervention.

Table S2. Characteristics of the health coaches (n=11)

No.	Age (years)	Gender	Occupation	Department or specialty	Full-time	No. of participants coached (M3)	No. of participants coached (Mfinal)
**C1**	35	F	Health management specialist	Medical Center (Health Management)	Y	47	89
**C2**	22	F	Undergraduate student	Neurology	N	10	NA‡
**C3**	22	M	Undergraduate student	Neurology	N	5	25
**C4**	55	F	Retired nurse	Neurology	Y	56	86
**C5**	41	F	Nurse	Internal Medicine	N	83	87
**C6**	58	F	Retired nurse	Internal Medicine	Y	24	44
**C7**	28	F	PhD candidate	Cognitive Disorders	N	9	9
**C8**	26	F	Master's student	Cognitive Disorder	N	11	11
**C9**	25	F	Master's student	Cognitive Disorder	N	9	9
**C10**	26	F	Master's student	Cognitive Disorder	N	7	7
**C11**	32	F	Nurse	Neurology	N	7	7

F – female, M – male, Y – yes, N – no, M3 – at approximately 3 months into the PRODEMOS intervention, Mfinal – at 12 to 18 months into the PRODEMOS intervention, PRODEMOS – PRevention Of DEmentia using MObile phone applicationS, NA – not applicable.
‡The coach quit at eight months after being involved in the PRODEMOS trial.
